# Racial/ethnic disparities in weight or BMI change in adulthood and pancreatic cancer incidence: The multiethnic cohort

**DOI:** 10.1002/cam4.3958

**Published:** 2021-05-16

**Authors:** Albert J. Farias, Samantha A. Streicher, Daniel O. Stram, Songren Wang, Stephen J. Pandol, Loïic Le Marchand, Veronica W. Setiawan

**Affiliations:** ^1^ Department of Preventive Medicine Keck School of Medicine University of Southern California Los Angeles CA USA; ^2^ Norris Comprehensive Cancer Center Los Angeles CA USA; ^3^ Epidemiology Program University of Hawaii Cancer Center Honolulu Hawaii USA; ^4^ Division of Gastroenterology Departments of Medicine Cedars‐Sinai Medical Center and Department of Veterans Affairs Los Angeles CA USA

## Abstract

**Introduction:**

Compared to non‐Hispanic Whites, Japanese Americans, Native Hawaiians, and African Americans have higher incidences of pancreatic cancer (PCa) that are not entirely explained by rates of obesity but may be explained by weight changes throughout adulthood.

**Methods:**

The multiethnic cohort is a population‐based prospective cohort study that has followed 155,308 participants since its establishment between 1993 and 1996. A total of 1,328 incident cases with invasive PCa were identified through 2015. We conducted separate multivariable Cox proportional hazards models for self‐reported weight‐change and BMI‐change (age 21 to cohort entry) to determine the association with PCa risk, adjusting for potential confounders including weight or BMI at age 21.

**Results:**

The mean age at cohort entry was 59.3 years (SD 8.9). An increased risk of PCa was associated with: 1) weight (HR per10 lbs = 1.06; 95% CI = 1.03–1.09) or BMI (HR per kg/m^2^ = 1.04; 95% CI = 1.02–1.05) at age 21; and 2) weight (HR per 10 lbs = 1.03; 95% CI = 1.01–1.05) or BMI (HR = 1.02; 95% CI = 1.00–1.03) at cohort entry. We found increased risk of PCa between weight (HR per 10 lbs = 1.03; 95% CI = 1.01–1.05) and BMI (HR per 5 kg/m^2^ = 1.08; 95% CI = 1.01–1.15) change from age 21 to baseline. There were significant interactions between race/ethnicity and weight (*p* = 0.008) or BMI (*p* = 0.03) at baseline, and weight (*p* = 0.02) or BMI (*p* = 0.02) change. Weight and BMI change through adulthood significantly increased the risk of PCa for Japanese Americans and Latinos, but not for African American, White, or Hawaiian participants.

**Conclusion:**

Our findings indicate that weight or BMI gain has a significant and independent impact on PCa risk, specifically among Latinos and Japanese Americans.

## Graphical abstract

In a large multiethnic prospective cohort, we found that weight and BMI change through adulthood was associated with an increased risk of pancreatic cancer. Weight or BMI gain is an independent risk factor for pancreatic cancer regardless of weight or BMI at age 21.

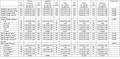

## 1 INTRODUCTION

Pancreatic cancer (PCa) is currently the fourth leading cause of cancer death^1^ in the United States, and it is projected to become the third leading cause of cancer death, surpassing lung and breast/prostate cancer, by 2022.^2^ Along with advancing treatment options after PCa diagnosis, identifying risk factors associated with PCa is crucial for early detection and potential preventive measures.^3^ Evidence suggests that risk factors for PCa include cigarette smoking, non‐O ABO blood group, chronic pancreatitis, long‐term diabetes mellitus, obesity, uncommon high‐penetrance germline mutations, and common low‐risk single nucleotide polymorphisms.^4^, ^5^, ^6^, ^7^, ^8^, ^9^, ^10^, ^11^, ^12^, ^13^

Epidemiologic evidence from observational studies, including meta‐analysis and pooled analysis, have consistently shown body mass index (BMI) in older adulthood to be a risk factor for PCa in a dose‐dependent manner.^14^, ^15^, ^16^, ^17^ When BMI in adolescence or early adulthood was examined, studies have also shown it to be associated with PCa.^16^, ^18^, ^19^, ^20^, ^21^, ^22^, ^23^ However, it remains unclear how adult weight and BMI gain influence PCa risk. While past studies of these variables have been more limited with few PCa cases, most have shown a non‐significant increased risk between greater weight or BMI change from adolescence/early adulthood to older adulthood and PCa.^19^, ^24^, ^25^

There are also striking racial/ethnic differences in PCa incidence in the United States that are not explained by rates of obesity.^26^ Native Hawaiians (RR: 1.60, 95% CI: 1.30–1.98), Japanese Americans (1.33, 1.15–1.54), and African Americans (1.20, 1.01–1.42) have a 60%, 33%, and 20% higher risk of developing PCa compared to non‐Hispanic Whites. While increased weight or BMI has been associated with risk of PCa within different populations, no individual study has compared the association between weight or BMI and risk of PCa among different racial/ethnic US populations.^23^, ^27^, ^28^

To further understand the relationship between weight or BMI and risk of PCa, the association between multiple weight or BMI measurements in adulthood and risk of PCa were examined in a diverse multiethnic cohort (MEC). The goal of this study was to examine the relationship between early adulthood weight and weight change (early adulthood and later in life) and incidence of PCa in the MEC, overall, and by sex and race/ethnicity.

## 2 METHODS

### 2.1 Study design and participants

A prospective cohort analysis was conducted among patients enrolled in the MEC. The MEC was established in 1993–1996 to investigate cancer etiology. It is comprised of over 215,000 participants between the ages of 45 and 75 at cohort entry, who were recruited from Los Angeles County and Hawaii. The five main ethnic groups represented in the MEC are White, African American, Latino American, Japanese American, and Native Hawaiian.^29^ All participants completed a self‐administered epidemiological baseline questionnaire, which included information on demographics, medical conditions, family history of cancer, and lifestyle factors. Individuals were excluded from this study if they were not of the five main race/ethnicity groups (N = 13,987), had missing BMI at baseline (N = 2,237), missing BMI at age 21 (N = 11,604), missing smoking status (N = 1,734), missing education status (N = 713), missing diabetes status (N = 2), missing alcohol intake (N = 6,948), missing vigorous physical activity (N = 7,166), invalid entry and exit dates (N = 6), had prevalent cancer at baseline (N = 14,914), had a reported BMI at baseline outside of 15–50 kg/m^2^ or had a BMI at age 21 outside of 15–50 kg/m^2^. In total, there were 155,308 eligible MEC participants for this analysis.

### 2.2 Independent study variables

The baseline epidemiological questionnaire assessed weight and height at cohort entry and asked respondents to recall weight at age 21. Body mass index (BMI) was calculated as weight (in kilograms) divided by height^2^ (in meters). In order to account for variations in the time that elapsed from age 21 to age at cohort entry, variables called weight change and weight change rate from age 21 to cohort entry and BMI change and BMI change rate from age 21 to cohort entry were created. Weight change from age 21 to cohort entry was created by subtracting weight at age 21 from weight at cohort entry. Weight change rate from age 21 to cohort entry was created by dividing the weight change from age 21 to cohort entry by the number of years elapsed between age 21 to cohort entry, which represents the average weight (lbs) change per year. In a similar manner, BMI change from age 21 to cohort entry was created by subtracting BMI at age 21 from BMI at cohort entry. BMI change rate from age 21 to cohort entry was created by dividing the BMI change from age 21 to cohort entry by the number of years elapsed between age 21 to cohort entry, which represents the average BMI (kg/m^2^) gain per year. Weight and BMI were modelled in four different ways: a continuous variable at age 21, a continuous variable at baseline, a continuous variable for change from age 21 to entry into MEC cohort, a continuous variable for change rate from age 21 to entry into MEC cohort and categories of weight/BMI change, similar to other studies.^30^, ^31^

### 2.3 Dependent study variable

PCa was modeled as a time‐dependent study end‐point with three possible outcomes: PCa diagnosis, censored at date of death or achieved end of study with no event. Annual linkages with the statewide Surveillance, Epidemiology and End Results (SEER) registries of Hawaii and California were used to identify incident cases of pancreatic cancer (ICD‐O‐3 codes C25.0‐C25.9). Only exocrine tumors were included in this analysis. Participants were censored if they died prior to the end of the follow‐up period based on linkages with death certificate files for Hawaii and California and the National Death Index. The date of last follow‐up was 31/12/2014 and the median follow‐up time was 20.2 years.

### 2.4 Covariates

We included covariates from data that were available from the baseline survey for age at cohort entry, sex (male, female, in overall, and race/ethnicity stratified models), race (African American, Japanese American, Latino, White, Native Hawaiian, in overall and sex stratified models), education (high school graduate or less, some college or technical school, college graduate, graduate and professional school), smoking status (never, former, current), alcohol intake (0, <12 g/day, ≥12 g/day), vigorous physical activity (hours/day), Health Eating Index 2010 (quartiles), diabetes at cohort entry (yes, no), and family history of pancreatic cancer (yes, no). The baseline dietary questionnaire was used to develop a Health Eating Index 2010 that captures the key nutrient and food group recommendations of the 2010 Dietary Guidelines and is used to assess the diet quality of the US population.^32^

### 2.5 Statistical analysis

Descriptive distributions were examined in the overall cohort, as well as stratified by weight change and BMI change from age 21 to cohort entry categories for descriptive purposes (>−10 lbs, −10 to +10 lbs, +10 to +30 lbs, +30 to +50 lbs, ≥50 lbs and <0 kg/m^2^, 0 to 5 kg/m^2^, 5 to 10 kg/m^2^, ≥15 kg/m^2^, respectively). The chi‐square test was used to compare categorical variables and the analysis of variance (ANOVA) test was used to compare continuous variables. multivariable cox proportional hazards (PH) analysis was used to calculate hazard ratios (HRs) and their 95% confidence intervals (95% CIs) as independent predictors of pancreatic cancer diagnosis. Models were adjusted for all potential confounders previously listed. Weight at age 21 (continuous) was adjusted for in the weight at baseline and weight change models. BMI at age 21 (continuous) was adjusted for in the BMI at baseline and BMI change models. The analyses were stratified by sex and race/ethnicity to assess whether the influence of absolute weight or BMI and weight or BMI change on pancreatic cancer risk varied by sex or racial/ethnic groups. We tested for an interaction by including a multiplicative variable to the regression model for the BMI and weight categories and race/ethnicity or sex. We also tested for significant interactions between smoking status and the weight or BMI variables using product terms. All statistical analysis was performed using SAS version 9.4 (North Carolina) and reported p‐values are based on two‐sided tests (summary alpha = 0.05). Values of *p* < 0.05 were used to define statistical significance. Smoking and diabetes status were found to violate the PH assumption in all models and were corrected by adding these variables to the STRATA command in SAS.

## 3 RESULTS

The main characteristics of the study population are presented in Table 1. Participants with a weight change of −10 to +10 lbs were more likely to be Japanese American or White, college or graduate/professional school graduates, never smokers, in the two higher quartiles of the Healthy Eating Index 2010 and have a family history of PCa. Participants with a weight change of ≥+50 lbs were more likely to be female, African American, Latino, or Hawaiian, high school graduates or have some technical school or college education, be former smokers, have no daily alcohol consumption, be in the lower two quartiles of the Healthy Eating Index 2010, and have pre‐existing diabetes. Among significantly different variables across BMI change groups from age 21 to entry into MEC, participants with a BMI change <0.9 kg/m^2^ were more likely to be older, Japanese American, college or graduate/professional school graduates, current smokers, engage in more vigorous daily physical activity, and in the fourth quartile of the Health Eating Index 2010, Participants with a BMI change ≥15 kg/m2 were more likely to be female, African American or White, high school graduates or have some technical school or college education, no daily alcohol consumption, in the third quartile of the Health Eating Index 2010, have pre‐existing diabetes and a family history of PCa (Table 1).

**TABLE 1:** Baseline cohort characteristics by change in weight and change in BMI from age 21 to entry into the Multiethnic Cohort (MEC)

Variable	Overall	Weight change from age 21 to entry into MEC						BMI change from age 21 to entry into MEC					
		>−10 lbs	−10 to +10 lbs	+10 to +30 lbs	+30 to +50 lbs	≥+50 lbs	*p* value	< 0 kg/m^2^	0 to 5 kg/m^2^	5 to 10 kg/m^2^	10 to 15 kg/m^2^	≥ 15 kg/m^2^	*p*‐value
No. subjects	155,308	5,614	26,951	56,212	37,889	28,642		14,168	78,546	46,556	12,037	4,001	
Age at enrolment / years	59.3 ± 8.78	61.3 ± 9.14	59.9 ± 9.18	59.3 ± 8.88	59.1 ± 8.56	58.5 ± 8.29	<0.0001	61.1 ± 9.18	59.2 ± 8.93	59.1 ± 8.50	58.5 ± 8.29	57.7 ± 8.09	<0.0001
Gender (%)							<0.0001						<0.0001
Male	47.4	52.1	45.5	49.4	49.0	42.0		48.4	52.2	45.7	30.5	18.5	
Female	52.7	47.9	54.5	50.6	51.0	58.1		51.6	47.8	54.3	69.5	81.5	
Race (%)							<0.0001						<0.0001
African American	15.7	13.2	8.3	11.6	18.0	28.0		10.3	11.4	19.3	28.9	36.8	
Japanese American	30.1	35.0	45.0	37.7	23.6	8.8		42.5	37.4	22.0	8.0	3.4	
Latino	21.8	17.7	13.2	19.8	27.0	27.8		14.9	18.2	27.9	29.1	24.8	
White	25.0	25.7	29.1	24.9	23.3	23.6		6.1	5.9	8.6	11.9	15.7	
Native Hawaiian	7.4	8.4	4.6	6.0	8.1	11.8		26.3	27.1	22.3	22.2	19.4	
Education (%)							<0.0001						<0.0001
High school graduate or less	42.0	47.3	36.6	39.8	44.3	47.6		42.8	38.0	45.8	50.0	50.4	
Some College or technical school	30.2	27.1	29.1	29.8	30.7	31.6		27.6	30.0	30.7	31.5	32.4	
College graduate	14.5	12.7	17.5	15.9	13.5	10.8		14.9	16.7	12.5	9.5	8.8	
Graduate and professional school	13.3	12.9	16.8	14.5	11.6	10.0		14.7	15.3	11.1	9.0	8.4	
Smoking status (%)							<0.0001						<0.0001
Never smoker	43.8	38.9	47.7	45.3	41.6	41.0		43.1	44.6	42.6	43.8	44.4	
Former smoker	40.0	35.4	33.8	38.8	43.1	44.8		34.6	38.8	42.8	42.4	42.4	
Current smoker	16.2	25.7	18.5	15.9	15.3	14.2		22.3	16.6	14.7	13.8	13.2	
Alcohol intake Category (%)							<0.0001						<0.0001
0	49.9	53.9	49.6	47.8	48.9	54.8		52.5	46.5	50.9	59.1	67.0	
< 12 g/day	30.6	24.9	29.5	31.5	31.6	29.6		26.7	31.5	31.2	28.8	24.6	
≥ 12 g/day	19.6	21.2	21.0	20.8	19.5	15.7		20.8	22.0	17.9	12.1	8.4	
Vigorous Activity / hours/day	0.40 ± 0.84	0.44 ± 0.94	0.44 ± 0.88	0.41 ± 0.83	0.38 ± 0.83	0.34 ± 0.80	<0.0001	0.43 ± 0.90	0.43 ± 0.86	0.36 ± 0.81	0.29 ± 0.73	0.22 ± 0.66	<0.0001
Health Eating Index 2010 (%)							<0.0001						<0.0001
Quartile 1 (53.7 points)	25.0	25.8	21.2	24.1	26.9	27.8		23.6	23.7	26.7	27.4	27.3	
Quartile 2 (63.4 points)	25.0	23.1	22.8	25.0	25.9	26.4		22.3	24.6	26.2	25.9	25.9	
Quartile 3 (70.8 points)	25.0	24.0	25.2	25.2	25.0	24.6		24.6	25.1	25.1	24.5	25.5	
Quartile 4 (79.9 points)	25.0	27.1	30.9	25.8	22.2	21.2		29.6	26.6	22.0	22.2	21.2	
Pre‐existing Diabetes (%)							<0.0001						<0.0001
Yes	11.1	16.7	7.6	8.9	11.6	16.8		11.9	8.5	12.5	17.8	22.6	
Family history of Pancreatic cancer (%)							<0.0001						0.0342
Yes	1.7	1.3	2.0	1.7	1.5	1.6		1.7	1.8	1.6	1.5	1.9	

*p*‐value is based on Chi‐squared test.

During the study period, 1,328 (0.86%) participants were diagnosed with PCa. The association between weight and BMI at age 21 or baseline and the risk of PCa, adjusted for possible confounding variables, are displayed in Table 2. Weight at age 21(HR per 10 lbs = 1.06, 95% CI=1.03–1.09), weight at baseline (HR per 10 lbs = 1.03, 95% CI = 1.01–1.05), weight change (HR = 1.03 per 10 lbs, 95% CI = 1.01–1.05), and weight change rate from age 21 to cohort entry (HR per lb per year = 1.12, 95% CI = 1.03–1.22) were associated with risk of PCa. Compared to weight change category −10 to +10 lbs, neither category <−10 lbs nor 10 to 30 lbs (HR = 0.94, 95% CI = 0.68–1.29 and HR = 1.02, 95% CI = 0.87–1.20, respectively) was associated with risk of PCa, but weight change category 30 to 50 lbs and ≥50 lbs (HR = 1.32, 95% CI = 1.11–1.56 and HR = 1.25, 95% CI = 1.03–1.52, respectively) were associated with risk of PCa. BMI at age 21 (HR = 1.04, 95% CI = 1.02–1.05), BMI at baseline/ (HR = 1.02, 95% CI = 1.00–1.03), BMI change (HR per 5 kg/m^2^ = 1.08, 95% CI = 1.01–1.16), and BMI change rate (HR per kg/m^2^ per year = 1.98, 95% CI = 1.20–3.26) were associated with risk of PCa. BMI change category 5–10 kg/m^2^ (HR = 1.23, 95% CI = 1.09–1.40) was associated with risk of PCa compared to a BMI change of 0 to 5 kg/m^2^ (Table 2). When the multivariable models were stratified by sex, weight change, BMI and BMI change variables appeared to be more strongly associated with risk of PCa among men than women; however, none of the interaction terms were significant with the exception for the interaction between the BMI rate change and sex (*p* = 0.0495) (Table 2).

**TABLE 2:** Cox Model for weight and BMI changes from age at 21 to entry into the MEC (N = 155308)

	PCa cases	Overall Model* N = 155,308	PCa cases	Male** N = 73,533	PCa cases	Female** N = 81,775	P interaction
		HR (95% CI)		HR (95% CI)		HR (95% CI)	
Weight at age 21/ 10 lbs	1328	1.06 (1.03–1.09)	656	1.05 (1.01–1.09)	672	1.07 (1.03–1.12)	0.5574
Weight at baseline/ 10 lbs	1328	1.03 (1.01–1.05)	656	1.05 (1.01–1.08)	672	1.01 (0.98–1.04)	0.4515
Weight change/ 10 lbs	1328	1.03 (1.01–1.05)	656	1.05 (1.01–1.08)	672	1.01 (0.98–1.04)	0.1833
Weight change rate/ lbs per year	1328	1.12 (1.03–1.22)	656	1.22 (1.08–1.37)	672	1.02 (0.91–1.15)	0.0812
Weight change Category							0.7708
<−10 lbs. (N = 5614)	49	0.94 (0.68–1.29)	27	0.99 (0.64–1.52)	22	0.88 (0.55–1.41)	
−10 to +10 lbs. (N = 26951)	224	1.00	108	1.00	116	1.00	
10 to 30 lbs. (N = 56212)	449	1.02 (0.87–1.20)	226	0.97 (0.77–1.22)	223	1.06 (0.84–1.33)	
30 to 50 lbs. (N = 37889)	364	1.32 (1.11–1.56)	182	1.26 (0.99–1.62)	182	1.34 (1.05–1.71)	
≥50 lbs. (N = 28642)	242	1.25 (1.03–1.52)	113	1.31 (0.99–1.74)	129	1.15 (0.87–1.52)	
P‐trend		0.0007		0.0097		0.0561	
BMI at age 21/ kg/m^2^	1328	1.04 (1.02–1.05)	656	1.03 (1.01–1.06)	672	1.04 (1.02–1.07)	0.7283
BMI at baseline / kg/m^2^	1328	1.02 (1.00–1.03)	656	1.03 (1.01–1.05)	672	1.01 (0.99–1.02)	0.2205
BMI change/ 5 kg/m^2^	1328	1.08 (1.01–1.16)	656	1.16 (1.04–1.30)	672	1.03 (0.94–1.12)	0.1322
BMI change rate/ kg/m^2^ per year	1328	1.98 (1.20–3.26)	656	3.87 (1.73–8.65)	672	1.21 (0.63–2.33)	0.0495
BMI change Category							0.2957
<0 kg/m^2^ (N = 14168)	123	0.93 (0.76–1.14)	66	1.05 (0.80–1.39)	57	0.81 (0.61–1.09)	
0 to 5 kg/m^2^ (N = 78546)	664	1.00	342	1.00	302	1.00	
5 to 10 kg/m^2^ (N = 46556)	437	1.23 (1.09–1.40)	211	1.29 (1.08–1.54)	226	1.15 (0.96–1.38)	
10 to 15 kg/m^2^ (N = 12037)	100	1.18 (0.95–1.47)	29	1.13 (0.77–1.67)	71	1.14 (0.87–1.50)	
≥15 kg/m^2^ (N = 4001)	24	0.89 (0.59–1.36)	8	1.59 (0.78–3.22)	16	0.69 (0.41–1.15)	
P‐trend		0.0201		0.0347		0.3151	

* Multivariate Cox model adjusting for age, sex, race, education, alcohol intake, height (Only for Weight‐related model), Health Eating Index 2010, smoking status, vigorous physical activity, pre‐existing diabetes, family history of pancreatic cancer and weight/BMI at 21 if appropriate.

** Multivariate Cox model adjusting for age at 21, race, education, alcohol intake, height (Only for Weight‐related model), Health Eating Index 2010, smoking status, vigorous physical activity, pre‐existing diabetes, family history of pancreatic cancer and weight/BMI at 21 if appropriate.

When the multivariable models were stratified by race/ethnicity, there were significant interactions between race/ethnicity and weight at baseline (*p* = 0.008), weight change (*p* = 0.02), and weight change rate (*p* = 0.02). The strongest associations for weight and weight change were seen in Japanese Americans and Latinos, with weight at baseline (HR per 10 lbs = 1.10, 95% CI = 1.05–1.15), weight change (HR per 10 lbs = 1.10, 95% CI = 1.05–1.15) and weight change rate/ (HR per lb per year = 1.41, 95% CI = 1.17–1.70) associated with risk of PCa in Japanese Americans and weight at age 21/10 lbs (HR = 1.09, 95% CI = 1.03–1.16), weight at baseline/10 lbs (HR = 1.07, 95% CI = 1.01–1.12), weight change/10 lbs (HR = 1.07, 95% CI = 1.01–1.12), and weight change rate (HR per lb per year =1.34, 95% CI = 1.11–1.61) associated with risk of PCa in Latinos. For Japanese Americans, there were also significant increases in risk of PCa for each increase in weight change category, compared to the reference weight change category of −10 to +10. For Latinos, compared to the reference weight change category of −10 to +10, each increase in weight category was associated with a larger non‐significant increase in risk of PCa (weight change >−10: HR = 0.59, 95% CI=0.23–1.49; −10 to +10: reference; 10 to 30: HR=0.83, 95% CI=0.52–1.32; 30 to 50: HR = 1.17, 95% CI = 0.74–1.84; HR = 1.30, 95% CI = 0.81–2.09) (Table 3).

**TABLE 3:** Cox Model for weight and BMI changes from age at 21 to entry into the MEC, by race (N = 155308) – Ns cat

	PCa cases	African American N = 24,324	PCa cases	Japanese American N = 46,744	PCa cases	Latino N = 33,852	PCa cases	White N = 38,834	PCa cases	Hawaiian N = 11,554	P interaction
		HR (95% CI)		HR (95% CI)		HR (95% CI)		HR (95% CI)		HR (95% CI)	
Weight at age 21/ 10 lbs	231	1.04 (0.98–1.11)	491	1.05 (0.99–1.12)	229	1.09 (1.03–1.16)	259	1.07 (1.00–1.13)	118	1.03 (0.96–1.12)	0.3152
Weight at baseline/ 10 lbs	231	0.98 (0.93–1.03)	491	1.10 (1.05–1.15)	229	1.07 (1.01–1.12)	259	0.98 (0.93–1.03)	118	1.01 (0.95–1.08)	0.0076
Weight change/ 10 lbs	231	0.98 (0.93–1.03)	491	1.10 (1.05–1.15)	229	1.07 (1.01–1.12)	259	0.98 (0.93–1.03)	118	1.01 (0.95–1.08)	0.0189
Weight change rate/ lbs per year	231	0.91 (0.76–1.08)	491	1.41 (1.17–1.70)	229	1.34 (1.11–1.61)	259	0.98 (0.81–1.17)	118	1.07 (0.88–1.32)	0.0206
Weight change category											0.2487
<−10	10	1.78 (0.80–3.99)	15	0.72 (0.41–1.25)	6	0.59 (0.23–1.49)	12	1.04 (0.54–2.00)	6	1.00 (0.37–2.70)	
−10 to +10	18	1.00	111	1.00	26	1.00	54	1.00	15	1.00	
10 to 30	50	0.93 (0.54–1.59)	229	1.26 (1.00–1.59)	61	0.83 (0.52–1.32)	79	0.79 (0.56–1.11)	30	0.72 (0.39–1.34)	
30 to 50	80	1.42 (0.84–2.39)	105	1.48 (1.12–1.94)	74	1.17 (0.74–1.84)	76	1.16 (0.81–1.66)	29	0.77 (0.41–1.46)	
≥ 50	73	1.14 (0.67–1.95)	31	1.71 (1.13–2.57)	62	1.30 (0.81–2.09)	38	0.80 (0.52–1.23)	38	0.94 (0.50–1.74)	
P‐trend		0.6347		0.0002		0.0177		0.8551		0.9031	
BMI at age 21/ kg/m^2^	231	1.03 (0.99–1.07)	491	1.03 (1.00–1.07)	229	1.05 (1.01–1.09)	259	1.04 (1.00–1.08)	118	1.02 (0.97–1.07)	0.7720
BMI at baseline / kg/m^2^	231	0.99 (0.96–1.01)	491	1.05 (1.03–1.08)	229	1.04 (1.01–1.07)	259	0.99 (0.96–1.02)	118	1.01 (0.97–1.05)	0.0330
BMI Change/ 5 kg/m^2^	231	0.93 (0.80–1.07)	491	1.30 (1.13–1.50)	229	1.20 (1.03–1.40)	259	0.94 (0.80–1.10)	118	1.04 (0.86–1.25)	0.0195
BMI Change rate/ kg/m^2^ per year	231	0.52 (0.17–1.56)	491	7.22 (2.49–20.9)	229	5.33 (1.74–16.4)	259	0.84 (0.26–2.68)	118	1.54 (0.43–5.55)	0.0249
BMI Change Category											0.0443
<0 kg/m^2^	14	1.16 (0.64–2.11)	48	0.66 (0.48–0.91)	17	1.15 (0.66–2.02)	33	1.35 (0.91–2.01)	11	1.19 (0.60–2.39)	
0 to 5 kg/m^2^	75	1.00	305	1.00	80	1.00	138	1.00	46	1.00	
5 to 10 kg/m^2^	106	1.39 (1.03–1.89)	127	1.28 (1.04–1.57)	99	1.49 (1.11–2.02)	70	1.01 (0.75–1.35)	35	0.88 (0.56–1.37)	
10 to 15 kg/m^2^	29	0.98 (0.63–1.53)	11	1.33 (0.73–2.43)	26	1.57 (1.00–2.47)	12	0.71 (0.39–1.29)	22	1.61 (0.96–2.72)	
≥15 kg/m^2^	7	0.60 (0.27–1.32)	0	–	7	1.61 (0.73–3.53)	6	1.31 (0.57–3.02)	4	0.73 (0.26–2.06)	
P‐trend		0.5665		0.0006		0.0206		0.2728		0.7457	

Note

Multivariate Cox model adjusting for age at 21, sex, education, alcohol intake, Health Eating Index 2010, height (Only for weight‐related model) smoking status, vigorous physical activity, pre‐existing diabetes, family history of pancreatic cancer and weight/BMI at age 21 if appropriate.

In the multivariable models stratified by race/ethnicity for BMI and BMI change, there were significant interactions between race/ethnicity and BMI at baseline (*p* = 0.03), BMI change (*p* = 0.02), BMI change rate (*p* = 0.05), and BMI change categories (*p* = 0.05). The strongest associations between BMI and BMI change and risk of PCa were also seen in Japanese Americans and Latinos, with BMI at age 21 (HR = 1.03, 95% CI = 1.00–1.07), BMI at baseline (HR = 1.05, 95% CI = 1.03–1.08), BMI change (HR=1.30, 95% CI = 1.13–1.50), and BMI change rate (HR per kg/m^2^ per year = 7.22, 95% CI = 2.49–20.9) associated with risk of PCa in Japanese Americans and BMI at age 21 (HR = 1.05, 95% CI = 1.01–1.09), BMI at baseline (HR = 1.04, 95% CI = 1.01–1.07), BMI change/ kg/m^2^ (HR = 1.20 95% CI = 1.03–1.40), and BMI change rate (HR per kg/m^2^ per year = 5.33, 95% CI = 1.74–16.4) associated with risk of PCa in Latinos. (Table 3). Compared to 0 to 5 kg/m^2^, for Japanese Americans (BMI change <0 kg/m^2^: HR = 0.66, 95% CI = 0.48–0.91; 5 to 10 kg/m^2^: HR = 1.28, 95% CI = 1.04–1.57; 10 to 15 kg/m^2^: HR = 1.33, 95% CI = 0.73–2.43) and Latinos (BMI change <0 kg/m^2^: HR = 1.15, 95% CI = 0.66–2.02; 5 to 10 kg/m^2^: HR = 1.49, 95% CI = 1.11–2.02; 10 to 15 kg/m^2^: HR = 1.57, 95% CI = 1.00–2.47; >15 kg/m^2^: HR = 1.61, 95% CI = 0.73–3.53) there were also upward trending HRs for each increasing BMI change category that were not seen in other racial/ethnic groups.

## 4 DISCUSSION

The relationships between weight or BMI at age 21 and at cohort entry and change from age 21 to cohort entry and risk of PCa were examined in the MEC, overall, and by sex and race/ethnicity. Overall, after controlling for potential confounding variables, our study shows a positive association between weight and BMI variables and risk of PCa. To our knowledge, this is the largest prospective cohort to examine weight or BMI change throughout adulthood and risk of PCa, the first study to examine racial/ethnic‐specific weight or BMI variables in a diverse racial/ethnic population, and the largest study to examine weight or BMI change variables among large cohorts of Japanese Americans, Native Hawaiians, and Latinos.

In our study, we did not observe significant differences in PCa risk by weight or BMI variables by sex. However, we did find that weight and BMI changes, change rate, and change categories were more strongly associated with PCa risk for men than for women. Nothings and colleagues^14^ examined the relationship between BMI in older adults and risk of PCa in an earlier analysis of the MEC study with follow‐up to 2002 and 237 cases and found that while the interaction between sex and BMI was not significant (*p* = 0.09), in men obesity (BMI≥30 kg/m^2^) was associated with an increased risk of PCa (HR = 1.51; 95% CI = 1.02–2.26), but in women it was associated with a reduced risk (HR = 0.65; 95% CI = 0.43–0.99).^14^ With 12 additional years of follow‐up and 1,091 more incident PCa cases in our current study, the association between weight or BMI variables and risk of PCa remained generally stronger in men than women, especially for weight or BMI change rate, but a reduced risk of PCa for women was no longer apparent. While several other studies have also revealed stronger relationships between weight or BMI variables in men compared to women, sex is not considered as an established modifier of the relationship between weight or BMI and risk of PCa, and the mechanism of this possible modification has not yet been explored.^23^, ^27^, ^28^, ^33^, ^34^

When we stratified our analysis by race/ethnicity, weight or BMI variables were positively associated with PCa risk among Japanese Americans and Latinos, but there was no consistent association for African Americans, Whites, or Native Hawaiians. The relative distribution of body fat is known to differ by race/ethnicity, with Latinos storing the highest amount as trunk fat and Japanese Americans storing the highest amounts as visceral fat, which may partially explain our differing findings by race/ethnicity.^35^ While is it unknown if the location of adiposity modifies the association between total adiposity and risk of PCa, several studies highlight the importance of exploring this potential modifier. For example, recent studies have shown that visceral fat is more strongly associated with risk of cancers such as colorectal and breast, compared to total adiposity.^36^, ^37^, ^38^

To our knowledge, our study is the first to examine weight or BMI change and risk of PCa in a large MEC study. Only one other large study in African Americans and one in Japanese have examined the relationship between absolute BMI in adulthood and PCa. A large pooled analysis of various African‐American studies that included 29,306 MEC subjects (140 PCa deaths), found a significant positive association between older adult BMI and death from PCa overall (P_trend_ = 0.03), but no significant association between older adult BMI and PCa death when limited to MEC subjects (HR = 1.11, 95% CI = 0.95–1.30).^27^ A large pooled analysis of Japanese in Japan showed an association between BMI in older adults and risk of PCa in females (HR per 1 kg/m^2^ = 1.02, 95% CI = 1.00–1.05), but not in males (HR per 1 kg/m^2^ = 0.97, 95% CI = 0.94–1.01).^23^ However, in our MEC study, we did not find a significant interaction between sex and BMI but we did find that BMI change from adulthood to cohort entry was significantly associated with PCa risk for males but not females. Further, we found that BMI and BMI change and weight and weight change was more strongly significantly associated with PCa risk among Japanese Americans than the other racial/ethnic groups included in our study.

The strengths of our analysis study include the large ethnically and racially diverse study population. There are some limitations to consider in interpreting these findings. First, weight at age 21 and baseline were self‐reported by subjects (not directly measured) and therefore subject to misclassification bias if participants did not accurately report weight at age 21. Second, although our cohort was large, the highest BMI change category (≥15 kg/m^2^), did not have enough subjects so the power to detect an association with risk of PCa is insufficient, especially when stratified by sex and race/ethnicity. Third, despite the fact that we controlled for a number of known and suspected confounders such as smoking history, history of diabetes, and family history of pancreatic cancer, there could still be residual or unmeasured confounding that we could not account for. Finally, there is a possibility of reverse causality due to disease‐related weight change, however, we conducted a sensitivity analysis excluding 118 patients diagnosed within three years of cohort entry and our findings remain unchanged.

In summary, our findings suggest that weight or BMI at age 21 and baseline and weight or BMI change are important risk factors for PCa. While there were no differences in weight or BMI variables when stratified by sex, there were significant disparities in weight or BMI variables when stratified by race/ethnicity, with only Japanese Americans and Latinos having positive associations between BMI or weight variables and risk of PCa.

## CONFLICT OF INTEREST

All authors have no potential conflicts to report.

## ETHICAL APPROVAL STATEMENT

Study protocols were approved by the Committee on Human Studies at the University of Hawaii and by the Institutional Review Board of the Keck School of Medicine of University of Southern California.

## Data Availability

Data are available on request from the authors.
